# INVERSE KLIPPEL-TRENAUNAY SYNDROME

**DOI:** 10.1590/1984-0462/2020/38/2020091

**Published:** 2020-12-18

**Authors:** Mariana Franco Ferraz Santino, Maria João Paiva Lopes

**Affiliations:** a Universidade Federal do Rio de Janeiro, Rio de Janeiro, RJ, Brazil.; b Centro Hospitalar Universitário de Lisboa Central, Lisboa, Portugal.

**Keywords:** Skin abnormalities, Vascular diseases, Subcutaneous tissue, Klippel-Trenaunay-Weber syndrome, Anormalidades da pele, Doenças vasculares, Tela subcutânea, Síndrome de Klippel-Trenaunay-Weber

## Abstract

**Objective:**

To report a rare case of inverse Kipplel-Trenaunay.

**Case description:**

A 16-year-old girl with a grayish-depressed plaque on her left thigh. Angioresonance showed a vascular malformation affecting the skin and subcutaneous tissue.

**Comments:**

Inverse Klippel-Trenaunay is a Klippel-Trenaunay syndrome variation in which there are capillary and venous malformations associated to hypotrophy or shortening of the affected limb. Modifications on the limb’s length or width result from alterations in bones, muscles, or subcutaneous tissues. It has few described cases. Further clinical and molecular studies must be performed for a proper understanding.

## RESUMO

**Objetivo:**

Relatar um caso raro de Klippel-Trenaunay inverso.

**Descrição do caso:**

Menina de 16 anos com placa deprimida acinzentada na coxa esquerda, evidenciando-se, por meio de angioressonância, uma malformação vascular, acometendo a pele e tecidos subcutâneos.

**Comentários:**

Klippel-Trenaunay inverso é uma variante da síndrome de Klippel-Trenaunay em que há malformação capilar e venosa associada à hipotrofia ou encurtamento do membro afetado. Pode envolver acometimento ósseo, muscular ou subcutâneo, modificando o comprimento ou a circunferência do membro. Há poucos casos descritos, e mais estudos clínicos e moleculares precisam ser realizados para seu correto entendimento.

## INTRODUCTION

Klippel-Trenaunay syndrome (KTS) is a rare vascular disorder present from birth or early childhood.^1^ ^,^ ^2^ It combines capillary and venous malformations with partial or total overgrowth of the involved limb.^1^ ^,^ ^2^ ^,^ ^3^ Its etiology is unknown.^1^ Inverse Klippel-Trenaunay syndrome (inverse KTS), in turn, is a new term for a paradoxical presentation in which the affected limb may become hypotrophic or shortened.^1^ Soft tissue volume reduction affecting the subcutaneous tissue, muscles, and bones has been reported.^4^ The present paper reports the rare case of a young female adolescent with vascular malformation associated to lipoatrophy on her left thigh.

## CASE DESCRIPTION

**Figure 1:**
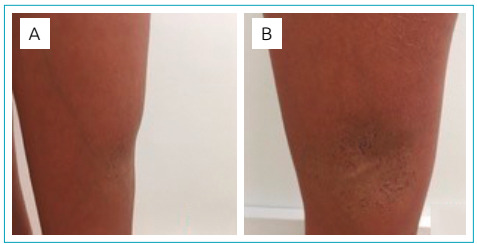
(A) Depressed plaque on the lower lateral face of the left thigh with visible vein reaching it; (B) grayish-depressed plaque, interspersed by hypochromic areas and with telangiectatic vessels located peripherally.

A 16-year-old female patient presented a 10.5 × 8.0 cm grayish-depressed plaque interspersed by hypochromic areas on the lower half of her left thigh lateral (Figure 1). Telangiectatic vessels overlap the lesion peripherally, with visible veins close to it (Figure 1). The plaque was present from birth, but it was violet initially. It evolved over the years, having whitened and depressed. There is no discrepancy in lower limbs’ length.

**Figure 2:**
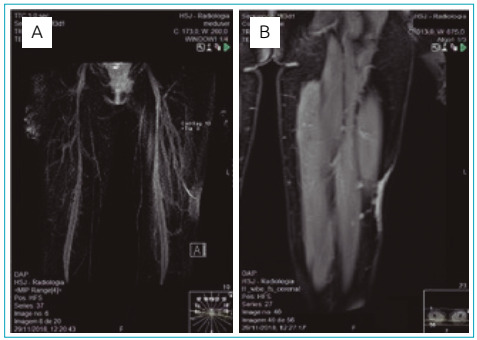
Vascular malformation on skin and subcutaneous tissue, supplied by non-dilated intermuscular branches tributary from popliteal artery, with corresponding thinning of the subcutaneous tissue thickness, but without extension to the musculature. (A) MIP reconstruction in coronal plane; (B) post-gadolinium sequence in coronal plane.

Angioresonance showed a vascular malformation on the skin and subcutaneous tissue supplied by non-dilated intermuscular branches tributary from the popliteal artery (Figure 2). Precocious venous filling was found both in the region and malformation, suggesting precocious venous shunt. Dilated drainage veins have not been documented, except for a superficial drainage vein that courses along the subcutaneous cellular tissue of the anterior thigh. In addition, a thinning of the subcutaneous tissue thickness in the vascular alteration topography was also noticed, but without intramuscular or bone extension.

## DISCUSSION

KTS is characterized by the presence of congenital capillary (*nevus flammeus* or port wine stain) and venous malformations (varicose veins and/or arteriovenous malformations) combined with bone and soft tissue hypertrophy on the extremities and adjacent parts of the trunk.^1^ ^,^ ^3^ ^,^ ^4^ ^,^ ^5^ Limb overgrowth is usually unilateral and may affect both length or width.^1^ ^,^ ^4^ ^,^ ^5^ Evolution is gradual, but at least one of the classical findings is already present at birth.^1^ ^,^ ^4^ ^,^ ^5^ Port wine stain may whiten over time and become a discolored area, according to studies.^2^ This is also our patient’s case.

Inverse KTS is rare and has few described cases.^1^ In a review by Danarti et al., 14 cases were evaluated as possible Inverse KTS.^5^ In these cases, the authors describe bone and/or soft tissue hypotrophy associated to the shortening or thinning of the involved limb.^1^ ^,^ ^4^ Most reports show associated muscular hypotrophy, but Capuccio et al. demonstrated a case in which the adjacent muscle had its structure and volume preserved,^2^ just like in the described patient.

The cause of deficiency in growth is unknown.^1^ Post-zygotic recombination of “minus” and “plus” alleles at the underlying gene locus is being investigated as a possible cause of hypotrophy.^3^ ^,^ ^4^ Soft tissue and bone involvement can be due to an unbalance in blood supply, resulting from a defective angiogenesis.^1^ More clinical and molecular studies are required for a deeper understanding.^1^ ^,^ ^2^
